# Mechanism of hypertension in diabetic nephropathy

**Published:** 2014-07-01

**Authors:** Chaudhary Muhammad Junaid Nazar

**Affiliations:** Department of Nephrology, Dialysis Center, Davita, Buridah Central Hospital, Saudi Arabia

**Keywords:** Hypertension, Diabetic nephropathy, Albuminuria

## Abstract

High prevalence of hypertension is observed in diabetic patients of both the types. Diabetic nephropathy is one of the major reason for high morbidity, mortality and financial burden in such hypertensive diabetic patients. For this review, electronic databases including PubMed/Medline, Embase, Cochrane and Google scholar were searched from 1990-2013. Multiple inter-related factors are responsible for the development of hypertension and therefore nephropathy in the chronic diabetic patients. Majority of such factors are identified to lead to extensive sodium reabsorption and peripheral vasoconstriction and thus leading to microvascular complications like nephropathy. Management of hypertension by targeting such mediators is the highly recommended therapy for controlling and treating diabetic nephropathy. Clinical trials suggests that drugs inhibiting the renin-angiotensin-aldosterone pathway should be used as the first-line agents for the management of hypertensive diabetic nephropathy patients. These agents are effective in slowing the progression of the end-stage kidney disease as well as lowering albuminuria. Researchers are also investigating the effectiveness of drug combination for better management of hypertension and diabetic nephropathy. The present article is a review of the evidences which explains the underlying pathological changes which leads to the development of nephropathy in a hypertensive diabetic patients. The review also observes the clinical trials for different anti-hypertensive drugs which are recommended for the treatment of such patients.

**Implication for health policy/practice/research/medical education:**

High prevalence of hypertension is observed in diabetic patients of both the types. Diabetic nephropathy is one of the major reason for high morbidity, mortality and financial burden in such hypertensive diabetic patients.

## Introduction

Prevalence rates of diabetes has been observed to increase drastically in past two decades due to both the ageing population around the world as well as unhealthy lifestyles which is increasing obesity and overweight problems. For this review, electronic databases including PubMed/Medline, Embase, Cochrane and Google scholar were searched from 1990-2013. Diabetic patients are always on a high risk of developing diabetes related complications like hypertension, neuropathy, nephropathy, retinopathy, stroke and others. Observations over-the-years suggest that one-third of the diabetic patient develops diabetic nephropathy which on long turn leads to chronic renal problems. Diabetic nephropathy is a one of the commonest problems responsible for diabetes related morbidity, mortality, and financial burdens. It is therefore essential to isolate the menace issues associated with the advancement of diabetic nephropathy. It is also highly advisable to have better knowledge of early treatment procedures so that extensive morbidity and mortality can be avoided (1).

A research suggests that diabetes mellitus and hypertension are highly interrelated and in majority of the cases predisposes the patient to atherosclerotic cardiac consequences. It is predicted that an excess of 3 million people in America are suffering from both blood sugar issues and hypertension. Prevalence of hypertension is doubles in the diabetes sufferers than without it. Although genetics is a leading factor in predisposing the patient concurrently to hypertension and diabetes; the simultaneous occurrence of both the conditions is hugely observed in the population of developed countries due to unhealthy lifestyle and eating habits. Observation of death certificates of over twenty thousand people in the United Kingdom implicated that 4.4% of the deaths occurred due to diabetes or the related complications out of which 10% were attributed to renal complications related to combined effect of diabetes and hypertension. Medical practitioners therefore always suggest early detection and aggressive of combined hypertension and diabetes (2).

Essential high tension level is widely observed in the hypertensive diabetic persons especially non-insulin dependent diabetes mellitus. These individuals constitute for 90% of the patients with dual diagnosis of hypertension and diabetes. Hypertension usually develops after chronic uncontrolled or under-controlled diabetes and then participates in the progress of diabetes nephropathy. This nephropathy normally develops after 15 years of dual exposure of diabetes and hypertension. Thirty percentile of the people with insulin-reliant blood sugar disease and 20 percentile of the people having non-insulin reliant blood sugar mellitus develops hypertension and therefore diabetic nephropathy in later years. Extensive association of hypertension with diabetes nephropathy is characterised by retaining of sodium concentrated fluids and as well as marginal vascular resistance (2).

The present paper attempts to extensively study the relationship between hypertension and diabetic nephropathy. The paper will include the underlying pathological observation as well as the impact of treatment modalities in the diabetic hypertensive patient with developing nephropathy.

## Definition and causes

Diabetic nephropathy is defined as macro albuminuria which is characterised by more than 300 mg of albumin excretion via urine within 24 h. Diabetic nephropathy is also observed as combined condition of macroalbuminuria as well as abnormal renal functioning which is observed as abnormal concentrates of serum creatinine, along with abnormality in calculated creatinine clearing and glomerular purification rates. In clinical settings, nephropathy of diabetes is diagnosed as advanced enhancement in urine protein defecation along other factors like reduced glomerular purification degree, hypertension, along with increased menace of heart related disturbances.

## Epidemiology

Hypertension is found in more than half of the sufferers having chronic blood sugar level in comparison to overall populace. Hypertension in diabetes mellitus type 1 leads to the development of microalbuminuria or overt nephropathy. The relationship between hypertensive diabetic patient and nephropathy is explained by a large Danish cross sectional study. The study observed a total of 1750 diabetic patients and 12,000 control people. It was found that the type 1 diabetic sufferer’s mellitus having no micro or macro-albuminuria, the occurrence rate of hypertension was not significantly different than the average populace (3.9% vs. 4.4%) (3). Another similar study by Lurbe observed similar study population but of a younger age than the previous study. The outcomes of the observational study suggested that pervasiveness of high tension is less in younger sufferers of diabetes and therefore the rates of nephropathy are also comparatively low. However, it was also found that a “non-dipping” nightly blood pressure occurrence even in a type 1 diabetic patient with usual urine albumin defecation may precipitate in to micro albuminuria and therefore can by identified as a probable high risk for the growth of renal problems like nephropathy (4). An early study also recognised the part of hereditary aspects in the development of microalbuminuria followed by microalbuminuria and thus nephropathy in hypertensive diabetic patients after extensively studying the family history of such patients (5).

For the type 2 diabetes mellitus patients, hypertension usually co-exist before the onset of kidney diseases. This can be explained by the fact that obesity and overweight problem is a common risk factor which is responsible for both the glucose intolerance as well as hypertension. Different studies suggest that prevalence of hypertension in the diabetic mellitus type 2 patients who are yet not having proteinuria is 58-70%. The research also clarifies that chronic diabetes is less associated with the development of hypertension than the impaired renal function. In fact, hypertension further exaggerate and worsen the dysfunction of kidneys and therefore directly contributes to exaggeration of the cardiovascular conditions (6).

The overall findings of the entire research suggest that microalbuminuria always precedes the hypertensive stage in both the types of diabetes patients and then the worsened renal functions contributes in degradation of the cardiovascular functions and the vicious cycle continues. The severity of high tension in diabetic nephropathy patient rises with every phase of the chronic kidney disease which in turn worsen the kidney functions and ultimately 90% of the patients are approached to final-phase renal ailment. An individual’s susceptibility to the development of both the high tension and renal ailment is caused due to various metabolic and hemodynamic changes which are shared by most diabetic patients. Genetic determinant are important decisive factors to dictate patient’s vulnerability. While certain inherited genes may make the person prone to the disease while some are renoprotective. Although this is yet unclear if these genetic factor defines the incidences of nephropathy diabetes or only make the person more vulnerable to the renal diseases in general context along with the other risk factors (7).

The international statistics for the prevalence of diabetic nephropathy reveals striking epidemiological variations even within the European nations. The proportion of the diabetic nephropathy sufferers requiring renal replacement treatment is more in Germany than the Unites States of America. Reports suggest that in the year 1995, 59% of the patients admitted in Southwest Germany hospitals for renal replacement were having any form of diabetes while 90% of them were having type 2 diabetes mellitus. A high correlation between final phase renal ailment and type 2 blood sugar has also been observed for the countries like Denmark and Australia where the overall prevalence of diabetes is low compared to the other states. Equal prevalence rate of the disease has been found in both the male and female patients. Epidemiological data of the condition in context of patient’s age suggest that diabetic nephropathy is rarely observed during the initial 10 year of the type 1 diabetes mellitus duration. On the other hand, peak incidences of the condition i.e. 3% per year has been found in the patients with diabetes for more than 15-20 years. Patients who usually requires management for final phase renal ailment are of average 60 years. While the reports from around the world suggest higher incidence of diabetic nephropathy in hypertensive diabetes sufferers of geriatric age, still the part of age in the progression of the ailment in younger sufferers is not clear. The Pima Indians having type 2 diabetes mellitus that are susceptible to the development of diabetes at younger age are also equally prone to the advancement of final phase renal ailment. Till a 20 year age, about fifty percent of every diabetic Pima Indian has acquired blood sugar nephropathy, where 15 percentile of these persons have advanced to end-stage renal disease (ESRD). Race based epidemiology of diabetic nephropathy reveals that the severity as well as occurrence rates of the condition are higher in the blacks who are around 3-6 times more affected by the condition than the whites, Mexican Americans, and Pima Indians who are having non-insulin reliant diabetic mellitus. The high prevalence of blood sugar nephropathy in black not only suggest the underlying genetic factors but also points out the effect of poor socioeconomic conditions which is the causative factor for poor diet, uncontrolled hyperglycemia, poorly managed hypertension and obesity. All these socioeconomic factors are also the threat aspects for the progress of diabetes, hypertension and thus blood sugar nephropathy. These facts also suggest that familial clustering may also be present in these population (8).

## Overall pathogenesis of diabetic nephropathy

The overall pathogenesis of blood sugar nephropathy is highly complex and is hugely driven by the altered internal milieu around the renal apparatus which initiates multiple pathways leading to the advancement of the ailment. Extensive hyperglycemia is a chief culprit in causing renal dysfunction as it leads to glomerular hyperfiltration along with endothelial dysfunction. Both the conditions together contributes to the changes in the basement membrane properties which are characterised by hypertrophy and hyperplasia of the intraglomerular cells. Other such basement membrane changes as observed by researchers are nodular intercapillary glomerulosclerose and glomerular matrix changes. Detection of albumin in the urine of patient is usually considered an indicative sign of such changes happening inside the body (9). All the anatomical changes are also associated with volume expansion and hypertension which occurs due to enhanced sodium reabsorption from the tubules. The proximal tubules uptake the protein and therefore increases the tubular protein load which in turn acts as a trigger to proinflammatory cascade which causes inflammation and fibrosis in the tubule-interstitial region. This entire process is accelerated if the patient is having chronic under controlled hyperglycemia (10).

The altered renin-angiotensin aldosterone system (RAAS) in the blood sugar patient also is a significant contributor to the advancement of blood sugar nephropathy. The altered RAAS system affects the systemic as well as glomerular blood pressure along with affecting the sodium reabsorption process by virtue of its angiotensin II and aldosterone effects on the pro-fibrotic level (11). Additionally the glycation of the renal tissue proteins due to prolonged hyperglycemia is even considered a vital issue for the progress of blood sugar nephropathy and microvascular complications. The excessive glucose in the hyperglycaemic patient bind to the circulating amino acids as well as tissue proteins which leads to certain non-enzymatic reaction which produces reversible glycation products which on further exacerbation produces advanced glycation endproducts also known as advanced glycation end products (AGEs). These AGEs are a stable compounds and are long lasting which gets in to the circulation and also gets deposited on the different tissues including kidneys (12). Furthermore, the interaction between the AGEs and their respective receptors triggers inflammatory cascade which on long run leads to endothelial dysfunction. All these findings thus suggest that identifying the accumulation of AGEs not only gives an idea of extensive hyperglycemia but also provides knowledge about the underlying metabolic burden, inflammatory processes, and oxidative stress due to the overload of superoxides and cellular pseudohypaxia. It is interesting to note that AGE accumulation is now getting acceptance as an early predictor of the forthcoming renal complication in the diabetic patients (13).

The observations that control of hypertension from the initial phases of blood sugar reduces the progression of blood sugar nephropathy suggest the important part of hypertension in the development of the condition. Prolonged systemic hypertension is an important contributor of the endothelial injuries to the kidneys. Human studies suggest that lowering the blood pressure with any of the pharmacological agent in a type 2 diabetes mellitus patient acts as a powerful intervention in the prevention of this complication (14).

**Figure 1:**
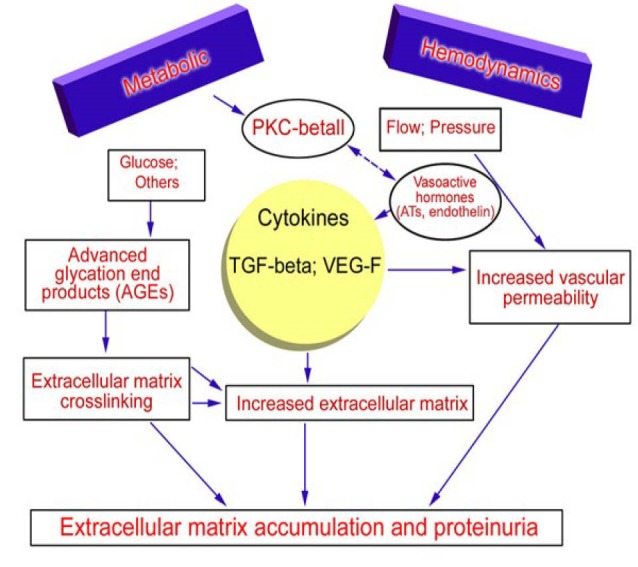
Pathogenesis of diabetic nephropathy (15).

The pathogenesis of diabetic nephropathy can be summarised by three distinctive mechanisms. 1) The expansion of mesangial membrane due to prolonged hyperglycemia which leads to heightened creation of matrix and/or glycosylation of the proteins in the matrix. 2) Coagulating of the glomerular cellar tissue because of various inflammatory processes. 3) Factors like expansion of the afferent renal artery or ischemic wound caused by the contracting of the blood vessels innervating the glomerular apparatus due to hyaline deposition (15). The major points of the pathogenesis are presented in a pictorial format in Figure 1.

## Genetic risk factors for diabetic nephropathy

Association of hereditary aspects in the growth and progression of blood sugar nephropathy can be explained by the fact that all the patients with either type of diabetes do not develops the condition. This suggests that inherited genetic factors in the family predisposes the person both to diabetes as well as diabetic nephropathy. According to a theory, a person born with comparatively lesser number of nephrons is highly susceptible to the growth of renal problems like nephropathy in the later life. Animal studies show that if the mothers are exposed to extensive hyperglycemia during pregnancy, the child is highly susceptible to the progress of diabetes in initial phases of life and also to the progress of final phase renal ailments. If this fact is extrapolated in case of humans, it suggest that certain maternal factors might also be responsible for the development of such complications (15).

Genetics along with the other risk factors like age, obesity, smoking, history of hypertension, and the length of the diabetic condition predisposes a diabetic patient to the development of diabetic nephropathy (16). Family-based linkage studies done on diabetic patients suggest that while some families are more susceptible to the advancement of blood sugar nephropathy; certain families even having a history of diabetes are well protected from the complication. Further investigations led to the identification of the susceptibility loci and its mapping on the various chromosomes in the humans (17). The specific gene mutations identified as the predisposing causes for the growth of blood sugar nephropathy are of ACE, NOS3, EPO, and APOL1. All these genes represents angiotensin converting enzymes, endothelial nitric oxide synthase, erythropoietin, and apolipoprotein L1. The chromosomal loci for these susceptible genes are mapped as 3q, 7q, 10p, 14q and 18q (18).

While the contribution of each of these genes in the progress of blood sugar nephropathy is not yet ascertained, extensive research in the field of diabetes related genetics may provide important information about the underlying pathophysiology of diabetic nephropathy as well as useful treatment targets in future. Two such genes which are presently extensively researched are CNDP1 and CCR2. Both the genes and their impacts are discussed below:

- CNDP1: The gene is an allelic variant of the carnosinase gene and has been strongly connected the development of blood sugar nephropathy by altering the carnosine pathway. The gene is responsible for encoding the enzyme carnosinase which functions as hydrolyzing enzyme to the substrate L-carnosine. Carnosine is also known as β-alanyl-L-histidine dipeptide and is found inside most of the cells in the body. Maximum amount of the amino-acid is found in the muscles and from there it is released inside the serum. The amino acid is also available from the nutritive sources. The main function of the carnosine is to inhibit the ACE in a natural environment and also hinder the production of the progressive glycation end-yields. The amino acid also reduces the oxidative stress by scavenging. Animal experimentation of the amino-acid as well as its laboratory cultures has proved that it hugely influence the breakdown of glucose in the physique and therefore reduces the risk of hyperglycemia. This in turn allows carnosine to protect the mesangial cells of the renal apparatus from the inflammatory effects and oxidative stress due to high blood glucose levels. In this manner carnosine prevents or at least slow down the progress of microvascular problems of blood sugar such as nephropathy (19). Extensive genetic research suggest that the genetic variant of carnosine gene with lower count of leucine is greatly effective in preventing the chances of nephropathy than those with higher number of leucine variants (20).
- CCR2: CCR2 encodes for chemokine receptor-2 gene. The chemokine receptor also acts as a co-receptor for macrophages and therefore plays an important role in inflammatory pathway. Mutation in the gene recognized as CCR-V64I is responsible for altering the monocyte chemoattractant protein-1 (MCP-1)-receptor and therefore induces the inflammatory processes under the influence of environmental conditions. The cascades initiated by such genes causes the development of inflammation mediated microvascular conditions like diabetic nephropathy (21).

## Prediction and diagnosis of diabetic nephropathy

While so many different menace issues for the blood sugar nephropathy was recognised, none of them are globally accepted as an accurate predictor of the condition. Presently the healthcare professionals use the signs of microalbuminuria as an early predictor of diabetic nephropathy. Albumin excretion as measured in the urine falling between 30 to 300 mg per day is considered as microalbuminuria. Also albumin creatinine ratio of 2.5-25 and 3.5-35 mg/mmol in men and female patients correspondingly is also considered as microalbuminuria. The ratio is identified from a random urine sample. These assessment not only predicts nephropathy but also indicates towards the progressive renal complications and therefore provides a prognosis related to mortality in both types of diabetes patients (22). While the international guidelines suggest the implementation of the microalbuminuria assessment as a screening modality for renal complications in any form of diabetes, the exact accuracy and precision of the test is yet debated. The role of the albumin testing for predicting renal complications is still not fully accepted in case of type 2 blood sugar mellitus patients as microalbuminuria might also be present because of generalised vascular damage. Microalbuminuria in type 2 diabetes patient not only suggest kidney damage but also indicates towards vascular damage to other vital organs like heart and the network of arteries (23).

Alternate non-invasive procedures for the early prediction of diabetic nephropathy are under investigation. Researchers have identified various urinary markers which acts as early predictors of renal complications. These new biomarkers are kidney injury molecule-1 (KIM-1), α-1 microglobulin and neutrophil gelatinase-associated lipocalin (NGAL). These biomarkers along with their practical application methods are further researched so that they can be used for prediction of both the acute as well as chronic renal damages caused due to diabetes (24).

Urine proteome analysis is another non-invasive technique which can be used for the early prediction of diabetic nephropathy. The method uses capillary electrophoresis coupled mass spectrometry to identify specific biomarkers in the patient’s urine sample (25). The proteome present in the urine consist of various stable as well as low molecular proteins which can be correlated with the progressive kidney complications like diabetic nephropathy. A study has identified 65 different biomarkers for diabetic nephropathy. All these biomarkers were also independently validated in a cohort population of 70 diabetic patients. The biomarkers accurately identified the blood sugar nephropathy patients having a sensitiveness and specification rate of 97% (26).

## Treatment of diabetic nephropathy

Various randomised control trial has proved that strict control of hyperglycaemic condition reduces the occurrence rate of albuminuria in the diabetic patients of both the types (27). It is necessary to note that once established, the albuminuria as well as lesions on the glomerular apparatus is hardly reversible even with the best glycaemic control. This suggest that even the most appropriate management of diabetes does not ensure limited progression of end-stage renal diseases if the albuminuria is already developed (28).

Inhibition of the renin-angiotensin aldosterone arrangement having an ACE inhibitor as well as angiotensin receptor blocker is presently considered as best treatment for preventing the development of overt blood sugar nephropathy to the final phase renal ailment. A relative risk reduction rate for reduced renal functioning as observed with the curing with ACE inhibitors in blood sugar sufferers is 50%. However the risk reduction with similar treatment plan was found to be only 15% for the final phase renal ailment for the non-insulin reliant blood sugar sufferers with fully established diabetes nephropathy (29).

Different strategies for effective blockade of RAAS have been proposed which even includes increased dosing of the drugs or even the utilisation of two or three different RAAS blocking drugs simultaneously. But such treatment plan implementation requires watchful observation of the patients hemodynamic balance as all these drugs when taken together may lead to hyperkalaemia in the patient (30).

All these data together suggest that even the rigorous management of hypertension and albuminuria has limited part in restricting the development of microvascular complications of blood sugar and thus adjunctive treatments are required which can directly affect the pathological pathway for the development of this conditions. Only pathway targeted treatment plans can effectively diminish the advancement of the diabetic nephropathy and added complications especially in type 2 diabetes mellitus patients.

Animal studies on benfotiamine found that the drug can prove to be an effective treatment option as it can act on the three major pathways of the hyperglycaemic damage. This sterol solvable thiamine derivative was proved to inhibit the hexosamine pathway, inhibit the forming of high level glycation final products, and can inhibit the diacylglycerol (DAG)-protein kinase C (PKC) pathway. The drug is also capable of blocking the initiation of the proinflammatory transcript issue NF-κB which is triggered by hyperglycemia. Instigation of the NF-κB conduit by hyperglycemia leads to the extensive production of triphosphates and superoxides which are responsible for the induction of metabolic pseudohypoxia. Ischemic damage caused by this pseudohypoxia is characterised by mitochondrial dysfunction and damage of renal cells by free oxygen radicals which ultimately leads to permanent vascular damage (31). Molecular studies suggest that both the thiamine and its derivative benfotiamine can inhibit these entire pathway by triggering the enzyme transketolase. The transketolase enzyme alters the glyceraldehydes-3-phospate and fructose-6-phosphate into pentose-5-phosphates and added sugars and therefore prevents the accumulation of glucose molecules due to hyperglycaemia in the tissues. In this manner the drugs restricts the progression of diabetes related vascular and microvascular complications including diabetic nephropathy. Animal studies also reported that higher doses of both the drugs were also able to prevent the development of microalbuminuria and proteinuria (32). Pilot level clinical trial of thiamine of three months duration on type 2 blood sugar sufferers having microalbuminuria suggested that the drug has a significantly constructive consequence on the reduction of albuminuria. However, further randomised control trials are required to predict whether benfotiamine is capable of decreasing albuminuria. It also needs to be tested for its impact on restricting or reducing the incidences of inflammatory cascade and fibrotic lesion by virtue of its effects on glucose intolerance and urinary albumin levels. If found beneficial and effective, the drug can be developed as a probable cure for restricting the progression of final phase renal diseases (33).

## Author’s contribution

CMJN was the single author of the paper.

## Conflict of interests

The author declared no competing interests.

## Ethical considerations

Ethical issues (including plagiarism, misconduct, data fabrication, falsification, double publication or submission, redundancy) have been completely observed by the author.

## Funding/Support

None.

## References

[1] Epstein M, Sowers JR (1992). Diabetes mellitus and hypertension. Hypertension.

[2] Rask-Madsen C, King GL (2010). Kidney complications: factors that protect the diabetic vasculature. Nat Med.

[3] Norgaard K, Feldt-Rasmussen B, Johnsen K, Saelan H, Deckert T (1990). Prevalence of hypertension in Type 1 (insulin dependent) diabetes mellitus. Diabetologia.

[4] Lurbe E, Redon J, Kesani A (2002). Increase in Nocturnal Blood Pressure and Progression to Microalbuminuria in Type 1 Diabetes. N Engl J Med.

[5] Fagerudd JA, Tarnow L, Jacobsen P, Stenman S, Nielsen FS, Pettersson-Fernholm KJ (1998). Predisposition to essential hypertension and development of diabetic nephropathy in IDDM patients. Diabetes.

[6] Freedman B, Bostrom M, Daeihagh P, Boweden D (2007). Genetic Factors in Diabetic Nephropathy. Clin J Am Soc Nephrol.

[7] Iynegar S, Freedman B, Sedor J (2007). Mining the genome for susceptibility to diabetic nephropathy: the role of large-scale studies and consortia. Semin Nephrol.

[8] de Boer IH, Rue TC, Hall YN (2011). Temporal trends in the prevalence of diabetic kidney disease in the United States. JAMA.

[9] de Boer IH, Rue TC, Cleary PA (2011). Long-term renal outcomes of patients with type 1 diabetes mellitus and microalbuminuria: an analysis of the Diabetes Control and Complications Trial/Epidemiology of Diabetes Interventions and Complications cohort. Arch Intern Med.

[10] Navarro-Gonzalez JF, Mora-Fernandez C, Muros de Fuentes M, Garcia-Perez J (2011). Inflammatory molecules and pathways in the pathogenesis of diabetic nephropathy. Nat Rev Nephrol.

[11] Anderson S, Vora JP (1995). Current concepts of renal hemodynamics in diabetes. J Diabetes Complications.

[12] Monnier VM (1990). Nonenzymatic glycosylation, the Maillard reaction and the aging process. J Gerontol.

[13] Amore A, Cirina P, Conti G (2004). Amadori configured albumin induce nitric-oxide dependent apoptosis of endothelial cells: a possible mechanism of diabetic vasculopathy. Nephrol Dial Transplant.

[14] Steffes MW (1999). Glomerular lesions of diabetes mellitus: preventable and reversible. Nephrol Dial Transplant.

[15] Batuman V. Diabetic nephropathy. 2012 [Online] Available from: http://emedicine.medscape.com/article/238946-overview#a0104.

[16] Adler S (2004). Diabetic nephropathy: Linking histology, cell biology, and genetics. Kidney Int.

[17] Vardarli I, Baier LJ, Hanson RL (2002). Gene for susceptibility to diabetic nephropathy in type 2 diabetes maps to 18q223-23. Kidney Int.

[18] Thomas MC, Groop PH, Tryggvason K (2012). Towards understanding the inherited susceptibility for nephropathy in diabetes. Curr Opin Nephrol Hyperten.

[19] Sauerhofer S, Yuan G, Braun GS (2007). L-carnosine, a substrate of carnosinase-1, influences glucose metabolism. Diabetes.

[20] Freedman BI, Hicks PJ, Sale MM (2007). A leucine repeat in the carnosinase gene CNDP1 is associated with diabetic end-stage renal disease in European Americans. Nephrol Dial Transplant.

[21] Wanic K, Placha G, Dunn J, Smiles A, Warram JH, Krolewski AS (2008). Exclusion of polymorphisms in carnosinase genes (CNDP1 & CNDP2) as cause of diabetic nephropathy in type 1 diabetes mellitus Results of large case - control and follow - up studies. Diabetes.

[22] Adler AI, Stevens RJ, Manley SE (2003). Development and progression of nephropathy in type 2 diabetes: the United Kingdom Prospective Diabetes Study (UKPDS 64). Kidney Int.

[23] Messent JW, Elliott TG, Hill RD, Jarrett RJ, Keen H, Viberti GC (1992). Prognostic significance of microalbuminuria in insulin-dependent diabetes mellitus: a twenty-three year follow-up study. Kidney Int.

[24] Waanders F, van Timmeren MM, Stegeman CA, Bakker SJ, van Goor H (2010). Kidney injury molecule-1 in renal disease. J Pathol.

[25] Julian BA, Wittke S, Novak J (2007). Electrophoretic methods for analysis of urinary polypeptides in IgA-associated renal diseases. Electrophoresis.

[26] Rossing K, Mischak H, Dakna M (2008). Urinary proteomics in diabetes and CKD. J Am Soc Nephrol.

[27] Intensive blood-glucose control with sulphonylureas
or insulin compared with conventional treatment and
risk of complications in patients with type 2 diabetes
(UKPDS 33). (2008). UK Prospective Diabetes Study (UKPDS) Group. Lancet.

[28] Ritz E, Rychlik I, Locatelli F, Halimi S (1999). End-stage renal failure in type 2 diabetes: A medical catastrophe of worldwide dimensions. Am J Kidney Dis.

[29] Brenner BM, Cooper ME, de Zeeuw Dl (2001). Effects of losartan on renal and cardiovascular outcomes in patients with type 2 diabetes and nephropathy. N Engl J Med.

[30] Parving HH, Persson F, Lewis JB, Lewis EJ, Hollenberg NK (2008). AVOID Study Investigators Aliskiren combined with losartan in type 2 diabetes and nephropathy. N Engl J Med.

[31] Brownlee M (2001). Biochemistry and molecular cell biology of diabetic complications. Nature.

[32] Babaei-Jadidi R, Karachalias N, Ahmed N, Battah S, Thornalley PJ (2003). Prevention of incipient diabetic nephropathy by high-dose thiamine and benfotiamine. Diabetes.

[33] Rabbani N, Alam SS, Riaz S (2009). High-dose thiamine therapy for patients with type 2 diabetes and microalbuminuria: a randomised, double-blind placebo-controlled pilot study. Diabetologia.

